# Evaluation of the Efficiency of Different Disruption Methods on Yeast Cell Wall Preparation for β-Glucan Isolation

**DOI:** 10.3390/molecules191220941

**Published:** 2014-12-15

**Authors:** Anna Bzducha-Wróbel, Stanisław Błażejak, Anna Kawarska, Lidia Stasiak-Różańska, Iwona Gientka, Ewa Majewska

**Affiliations:** 1 Department of Biotechnology, Microbiology and Food Evaluation, Warsaw University of Life Sciences-SGGW, Nowoursynowska 159c, Warsaw 02-776, Poland; E-Mails: stanislaw_blazejak@sggw.pl (S.B.); kawarska.anna@gmail.com (A.K.); lidia_stasiak_rozanska@sggw.pl (L.S.-R.); iwona_gientka@sggw.pl (I.G.); 2 Department of Chemistry, Warsaw University of Life Sciences-SGGW, Nowoursynowska 159c, Warsaw 02-776, Poland, E-Mail: ewa_majewska@sggw.pl

**Keywords:** β-glucan, yeast cells wall, bead mill, autolysis, sonication, autoclaving

## Abstract

Selected methods for yeast cell disruption were evaluated to establish their suitability for cell wall preparation in the process of β-glucan isolation. The effect of different disruption methods on contents of total saccharides, β-glucans and proteins in the produced cell walls preparations was analyzed. The degree of cell wall purification from intracellular components was established on the basis of the ratio of solubilised material. The investigated methods included: cell exposure to hot water (autoclaving), thermally-induced autolysis, homogenization in a bead mill, sonication and their combinations. Experimental systems were prepared in water (pH 5.0 and pH 7.0) and Tris-HCl buffer (pH 8.0). The *Saccharomyces cerevisiae* yeast cell wall preparations with the highest degree of cytosol component release and purification of β-glucans were produced by 30 min of cell homogenization with zirconium-glass beads (0.5 mm in diameter). This was confirmed by the highest ratio of solubilised material (approx. 64%–67%). The thus-produced preparations contained *ca.* 60% of total saccharides, 13%–14% of β(1,3)/(1,6)-glucans, and approx. 35% of crude proteins. Similar results were obtained after autolysis coupled with bead milling as well as with sonication, but the time required for these processes was more than 24 h. Homogenization in a bead mill could be valuable for general isolation procedures because allows one to eliminate the different autolytic activity of various yeast strains.

## 1. Introduction

β-(1,3)/(1,6)-Glucans are the main structural polysaccharides of the cell walls of yeast and they have confirmed therapeutic properties [1,2]. They display immunostimulating effects, including anti-microbial, anti-inflammatory, anti-carcinogenic effects and they accelerate wound healing [2,3,4,5,6,7,8,9]. In 2011, the European Commission issued a decision on yeast β-glucan inclusion to the list of novel food components according to the Regulation no. 258/97 of the European Parliament. In agreement with this document, yeast β*-*glucans may be applied in specified categories of food products, including as components of functional food and food of special nutritional designation (EC Decision of 24 November 2011). Owing to their technological functionality (e.g., as water- and lipid-binding components, gelling substances, emulsion stabilizers, thickening agents), these polysaccharides may, apart from the food industry, also be applied in the production of cosmetics. In the latter case, β*-*glucans’ capability to stimulate collagen synthesis and of their antioxidative properties which prevent skin ageing are also important [2].

Biotechnological production of β(1,3)/(1,6)-glucans is based on their isolation from yeast biomass, usually these of the species *S. cerevisiae*. However, there are also reportss on studies focused on the cell wall polymers of yeast originating from other taxonomic groups, which may contribute to the identification of polymers with novel properties [9,10].

The location of β-glucans in the structure of a cell wall requires cell disruption to enable the production of cell wall preparations and then the glucose polymer isolation. β-Glucans is more easily extracted from cell walls than from intact cells [11]. Yeast cell walls are relatively rigid structures due to the β(1,3)-glucans [12,13]. Release of intracellular molecules is determined by the functionality of the plasma membrane and the porosity of the yeast cell wall [14]. Efficient breakage of cell wall strength-providing components is necessary to effectively remove intracellular compounds [12]. Microbial cell disruption methods can be mainly classified into mechanical and non-mechanical ones [12]. Mechanical methods, like bead milling, sonication or high-pressure homogenization, are based on shear forces. Non-mechanical procedures are divided into physical (e.g., thermolysis, osmotic shock), chemical and enzymatic (including autolysis) methods. Various quantitative measures of the efficiency of disruption may be applied. For example, direct microscopic methods allow one to estimate the number of cell lysed or disintegrated while indirect methods measure the release of specific intracellular components resulting from biomass solubilization [13]. Also physical properties of disrupted cells (size of cell debris, particle size distribution, viscosity) could be taken into consideration [15]. Transmission electron microscopy (TEM) or scanning electron microscopy (SEM) are used to observe the alteration of yeast cells after different disruption processes. Hernawan and Fleet [16] established, using TEM and SEM, that the cell walls of different yeast retain their basic shape, surface appearance and integrity during autolysis. This was probably because the core β(1,3)-glucans were not degraded. According to the mentioned authors [16], the loss of cell wall mannoproteins would not affect wall integrity, but rather alter the wall porosity which facilitates the release of intracellular macromolecules. Liu* et al*. [17] noticed, based on SEM and TEM, that after autolysis the separation of yeast cell walls from cytoplasm, occurred but the cell walls were still intact. Autolysis thus did not impair cell walls but was helpful to discard unwanted intracellular substances during preparation of cell walls. Hot water treatment caused a significant swelling of the cell wall while cells were much smaller in size. After 1 min of yeast cell maceration using a bead mill Ŕeháćek* et al*. [18] observed that the cell walls were mostly intact. The desired degree of fragmentation was achieved after prolonging the milling time. The tridimensional shape of yeast cell walls is lost after sequential enzymatic lysis by proteases and glucanases which release wall proteins, attached mannans and solubilize β-glucans, respectively [13]. The purity of the isolated cell walls could be determined microscopically and chemically [18].

The literature reports various methods for yeast cell disruption during production of β-glucans [7,9,17,19,20,21,22,23,24,25,26,27,28,29]. However, data on the efficiency of individual stages of the process that would consider the purity of the resultant preparations or losses of isolated components is usually lacking. Long-lasting autolysis is most often used [7,17,24,28,29]. On the other hand, most of the published articles concerning microbial disruption were focused on isolation of intracellular products of bacteria or fungi and not on cell wall preparations [30]. According to our knowledge, the literature is lacking results that compare and evaluate the suitability of different methodologies for the preparation of yeast cell walls utilized for β*-*glucan isolation. This prompted authors of this study to verify selected methods of yeast disruption in the process of producing cell wall preparations. In this study we examined the effect of hot water extraction (autoclaving), thermally-induced autolysis, disruption with the use of a bead mill, sonication and combinations of these methods on the purity of the produced preparations. The goal of this analysis was to identify a method or methods that allow producing preparations with possibly the highest content of saccharides and β*-*glucans to ensure lower inputs of reagents and energy in the successive stages of purification from proteins and lipids.

## 2. Results and Discussion

### 2.1. The Influence of the Tested Methods on the Solubilization of Yeast Cell Biomass

The studied methods of yeast cells disruption decreased the dry weight of yeast biomass. Table 1 presents results of the effect of the evaluated methods of yeast cell disruption on the percentage of solubilised *S. cerevisiae* biomass material after the disruption processes. The lower level of yeast cell material solubilization, indicative of the poorest effectiveness of yeast cells disruption, was determined after autoclaving (approx. 18%). The highest percentages of yeast solubilised material were noted after cell disruption using homogenization in a bead mill and autolysis coupled with bead milling. The mentioned methods enabled achieving the solubilisation of yeast material in the 61%–67% range. Thus, these were the most effective methods for yeast cell disruption and purification from cytosol components. However, Hernawan and Fleet [16] stated that during yeast autolysis the yeast cell wall polysaccharides were actually not degraded.

**Table 1:** Effect of the analyzed methods of yeast cells disruption on the ratio of solubilised material, contents of total saccharides, β(1,3)/(1,6)-glucan and crude proteins in *S. cerevisiae* yeast cell wall preparations.

Experimental System	Solubilised Material (%)	Total Saccharides	β(1,3)/(1,6)-glucan	Crude Proteins
		$\bar{x}$ ± SD (g/100 g _d.m._ Preparation)		
Biomass	-	42.5 ± 2.0 ^A,B^	7.7 ± 0.3 ^B^	45.3 ± 2.6 ^F^
a *	18.4 ± 0.6 ^A,^**	47.0 ± 3.7 ^A,B,C,D^	5.7 ± 0.1 ^A^	53.6 ± 1.5 ^F,G^
a_s	25.5 ± 0.5 ^B^	39.4 ± 5.6 ^A^	6.1 ± 0.4 ^A^	54.3 ± 0.6 ^F,G^
a_m	30.5 ± 0.5 ^D^	41.5 ± 4.8 ^A^	6.4 ± 0.4 ^A^	55.8 ± 1.1 ^G^
aB	27.8 ± 0.3 ^C^	44.8 ± 3.4 ^A,B,C^	5.7 ± 0.3 ^A^	51.5 ± 0.5 ^F^
a_sB	26.3 ± 0.5 ^B,C^	45.0 ± 0.9 ^A,B,C^	5.8 ± 0.4 ^A^	54.2 ± 0.3 ^F,G^
a_mB	41.4 ± 0.4 ^E^	43.7 ± 1.6 ^A,B^	6.6 ± 0.1 ^A^	56.0 ± 0.6 ^G^
al_pH7	55.6 ± 1.2 ^H,I^	57.2 ± 1.4 ^D,F,G,H^	12.9 ± 0.4 ^D,E^	35.0 ± 0.1 ^A^
al_pH5	53.5 ± 0.8 ^G,H^	63.5 ± 1.7 ^G,H^	12.5 ± 0.4 ^D^	35.2 ± 0.7 ^A^
al_s	52.6 ± 0.8 ^F,G^	60.5 ± 1.2 ^F,G,H^	15.5 ± 0.4 ^G^	36.5 ± 0.5 ^A,B^
al_m	62.9 ± 0.5 ^K,L^	61.2 ± 1.7 ^F,G,H^	14.5 ± 0.6 ^F,G^	36.7 ± 0.6 ^A,B^
alB	56.1 ± 0.3 ^I^	64.4 ± 1.5 ^H^	12.0 ± 0.2 ^C,D^	36.2 ± 0.7 ^A,B^
al_sB	60.5 ± 0.5 ^J^	62.5 ± 2.9 ^F,G,H^	12.2 ± 0.2 ^C,D^	36.6 ± 0.3 ^A,B^
al_mB	60.7 ± 1.5 ^J^	57.3 ± 1.6 ^E,F,G,H^	11.2 ± 0.2 ^C^	42.9 ± 0.4 ^D,E,F^
s	33.2 ± 0.4 ^D^	47.9 ± 2.1^A,B,C,D,E^	6.4 ± 0.2 ^A^	44.3 ± 0.1 ^E,F^
sB	52.7 ± 0.6 ^G^	58.8 ± 3.0 ^F,G,H^	12.9 ± 0.3 ^D,E^	40.8 ± 0.4 ^C,D,E^
m 0.5	64.4 ± 0.8 ^L,M^	59.7 ± 2.8 ^F,G,H^	13.8 ± 0.6 ^E.F^	34.8 ± 0.8 ^A^
m 1.0	61.2 ± 0.3 ^J,K^	56.3 ± 2.3^C,D,E,F,G,H^	12.1 ± 0.5 ^C,D^	37.8 ± 0.9 ^A,B,C^
m 0.5B	66.5 ± 0.8 ^M^	53.1 ± 3.4 ^B,C,D,E,F^	14.1 ± 0.6 ^F^	37.4 ± 0.8 ^A,B,C^
m 1.0B	61.5 ± 0.5 ^J,K^	54.0 ± 5.1 ^C,D,E,F,G^	12.4 ± 0.3 ^D^	39.6 ± 0.6 ^B,C,D^

Notes: * a—autoclaving; al—autolysis; s—sonication; m—bead milling; B—buffer. Descriptions of the abbreviations as in Table 2 (Experimental Section); ** A, B, C, D, E, F, G, H —mean values in columns denoted with the same letter are not significantly different (Tuckey’s test, α = 0.05).

The dry weight of yeast biomass is composed mainly of proteins (45%–50%), while the nucleic acid content ranges from 5% to 15% on a dry weight basis in the case of RNA and approx. 0.1%–1.5% considering DNA [16,31]. Protein release is one of a key characteristic of microbial cell disruption [13,14].

Based on literature data [12], the efficiency of the tested methods in releasing intracellular yeast compounds was additionally evaluated by reading the absorbance value of the supernatant solutions of yeast-solubilised material at wavelengths λ = 260 nm and λ = 280 nm. The absorbance peaks at around the mentioned wavelengths correspond to absorption by nucleic acid and proteins, respectively, which are the main intracellular constituents of *S. cerevisiae*. However, nucleic acid interference in some cases extends the absorbance at 280 nm while phenylalanine increases the values at 260 nm.

**Figure 1:**
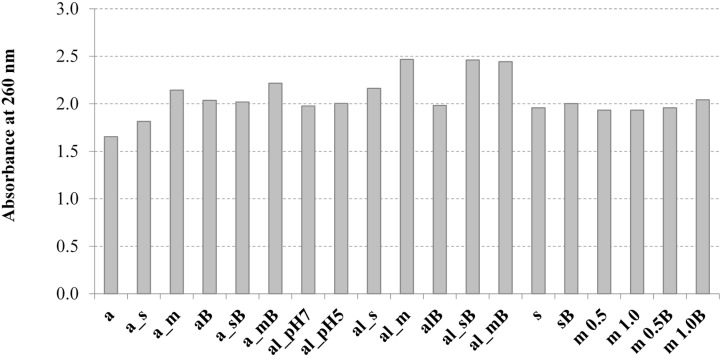
Effect of tested disruption methods on the absorbance at 260 nm of solubilised material of yeast biomass; a—autoclaving; al—autolysis; s—sonication; m—bead milling; B—buffer. Descriptions of the abbreviations as in Table 2 (Experimental Section).

**Figure 2:**
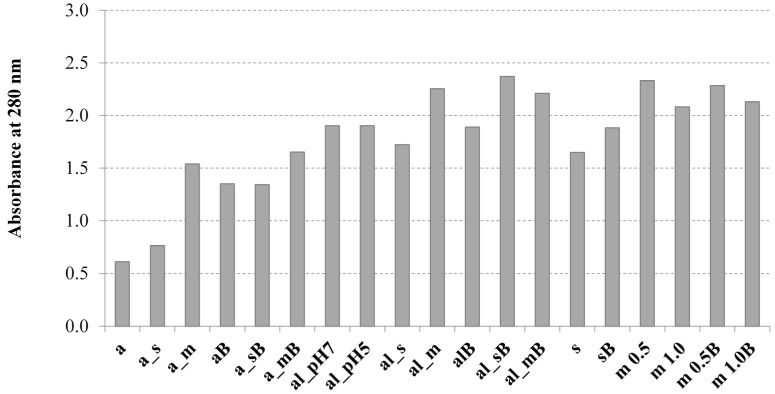
Effect of tested disruption methods on the absorbance at 280 nm of solubilised material of yeast biomass. a—autoclaving; al—autolysis; s—sonication; m—bead milling; B—buffer. Descriptions of the abbreviations as in Table 2 (Experimental Section).

The results of absorbance at 260 nm and 280 nm (8-fold diluted samples) are presented in Figure 1 and Figure 2, respectively. The obtained data confirmed the release of intracellular genetic material and proteins during the tested disruption processes. The highest absorbance values at 260 nm (Figure 1) were observed for autolysis combined with bead milling or with sonication. Zhao and Fleet [31] confirmed the extensive nucleic acid degradation (mainly RNA) during autolysis.

Hot water extraction, which took place during autoclaving, was characterized by the lowest efficiency of nucleic acids release. At the same time, the hot water extraction process solubilized the genetic material more effectively compared with cellular proteins (Figure 2). Middelberg [13] explained that for samples containing partially denaturated material the solubility of proteins changed with dilution.

Analysis of the results obtained after measurement of sample absorbance at 280 nm (Figure 2) allowed us to state the more diversified influence of the studied disruption methods on protein solubilization of yeast biomass compared with nucleic acids. The highest level of protein release was noted after yeast cell autolysis coupled with mechanical methods like bead milling and sonication. Homogenization in a bead mill as a single process was also effective. Autoclaving was characterized by the lowest level of protein solubilization.

### 2.2. Effect of the Analyzed Disruption Methods on Contents of Total Saccharides, β(1,3)/(1,6)-Glucans, and Proteins in S. Cerevisiae Cell Wall Preparations

Among all analyzed methods of yeast disruption, the *S. cerevisiae* cell autoclaving turned out to be the least effective method for the production of cell wall preparations. In this case, the contents of total saccharides were similar to these reported in the biomass of intact cells (Table 1), regardless of the type of medium used in the disruption process (deionized water or buffer). The coupling of autoclaving with sonication or bead mill homogenization did not increase the effectiveness of this method either. Characteristic for this part of results was the fact that the crude protein content in all preparations produced upon autoclaving was higher (*ca.* 51%–56%) than the initial content of this component in yeast biomass (*ca.* 45%). The analysis of yeast cell disruption effectiveness after autoclaving, determined based on microscopic observations of the cell wall preparations, enabled us to conclude that all cells had been killed, however only a few of them lost their cell wall continuity. This resulted in an insignificant release of the cellular content to the medium. The higher content of proteins in all cell walls preparations produced via autoclaving, compared to the dry biomass of yeast, could be due to retention of intracellular proteins and other components with simultaneous extraction of cytosolic components including sugars and genetic material, but also cell wall components soluble in water, like mannoproteins and β(1,6)-glucan. In consequence, the protein content in the preparation was increasing. The retention of the yeast cells suspension in the autoclave caused partial loss of structural polymers of the cell wall soluble in hot water as a result of β-glucans extraction. This was confirmed by the lower content of β(1,3)/(1,6)-glucans in yeast cell wall preparations produced via autoclaving compared to yeast biomass not subjected to disruption (Table 1). The losses reached *ca.* 13.5%–26.0%, however the greatest losses were reported using only autoclaving in water or buffer. Further research is needed to determine the sugars present in the supernatants to ensure an accurate mass balance.

The cell wall preparations produced via yeast autolysis were characterized by a content of total saccharides of *ca.* 57% to 64% (Table 1). This data points to significant purification of the preparations from cytosol components, which was confirmed by a protein content reduction by *ca.* 33% in the yeast biomass. It was also found that the pH value of water used to prepare yeast suspension (pH 5.0 and 7.0) had no significant effect on the degree of cell autolysis. Considering that, experimental systems with coupled methods were only tested at pH 5.0.

After autolysis in water, the content of β-glucans in the cell wall preparations was higher compared to that determined in the preparations after cell lysis in Tris-HCl buffer. Harnawan and Fleet [16] confirmed that during autolysis the cellular polysaccharides associated mainly with cell walls were only weakly degraded. Presumably, during 24 h autolysis in buffer system (pH 8.0), the alkali-soluble β-glucans fraction could be extracted.

**Figure 3:**
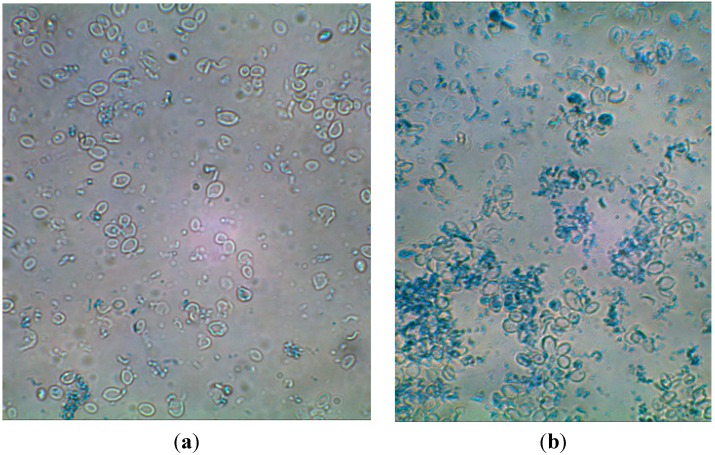
*S. cerevisiae* cell wall preparations after cells disruption with the use of autolysis and bead mill (magn. 600×); (**a**) cells disrupted in deionized water; (**b**) cells disrupted in Tris-HCl buffer.

**Figure 4:**
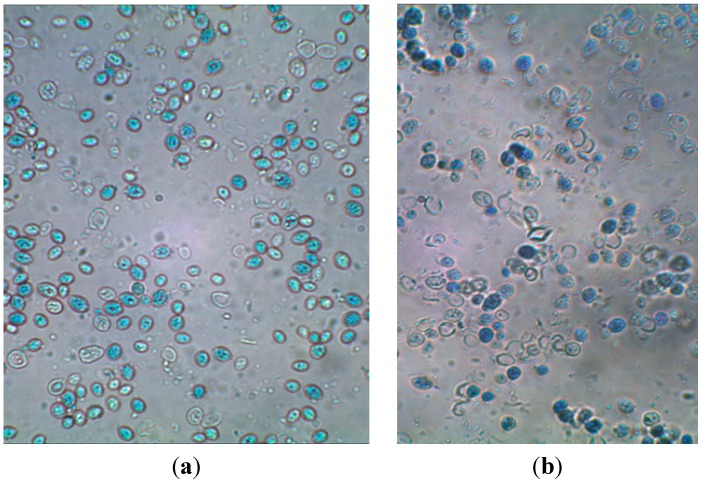
*S. cerevisiae* cell wall preparations after cells disruption with the use of autolysis and ultrasounds (magn. 600×); (**a**) cells disrupted in deionized water; (**b**) cells disrupted in Tris-HCl buffer.

The highest degree of purification of cell wall preparations was determined after yeast cell autolysis in water followed by homogenization in a bead mill or by sonication. In both cases, the content of β(1,3)/(1,6)-glucans in the cell wall preparations (14.5%–15.5%) was *ca.* twofold higher than their content in biomass (7.7%). The effective purification of the cell wall preparations produced via autolysis could be obviously due to the hydrolytic activity of endogenous proteolytic enzymes directed at intracellular proteins, whereas disruption with a bead mill resulted in the best fragmentation of cells, which contributed to the effective release of cytosol components. Figure 3 presents exemplary photos of cells after autolysis coupled with homogenization in a bead mill. Disrupted and disintegrated cells are observed, deprived of intracellular components. The discussed method of disruption caused more intensive fragmentation of cells compared to ultrasound (Figure 4).

Disruption of *S. cerevisiae* yeast cells with the use of fast (app. 30 min) homogenization in a bead mill (as an individual method), produced a comparable degree of purification of cell wall preparations compared to 24 h autolysis. In the case of yeast bead milling in water, the content of total saccharides in the cell wall preparation was 59%–60% and that of proteins 35%–38% d.m. (Table 1). The use of homogenization with beads having diameters of 0.5 or 1.0 mm produced very similar effects. An insignificantly higher degree of purification from proteins (*ca.* 2%) was achieved by using 0.5 mm diameter beads. It was reflected in a minimally higher content of β-glucans in the cell wall preparations after homogenization using beads with the smaller diameter (by *ca.* 14 and 12%, respectively). The effectiveness of purification of cell wall preparations with the use of a bead mill as an individual method was unaffected by the type of medium applied to prepare the yeast suspension. Contents of total saccharides, proteins and β-glucans in all preparations produced with this method did not differ significantly.

**Figure 5:**
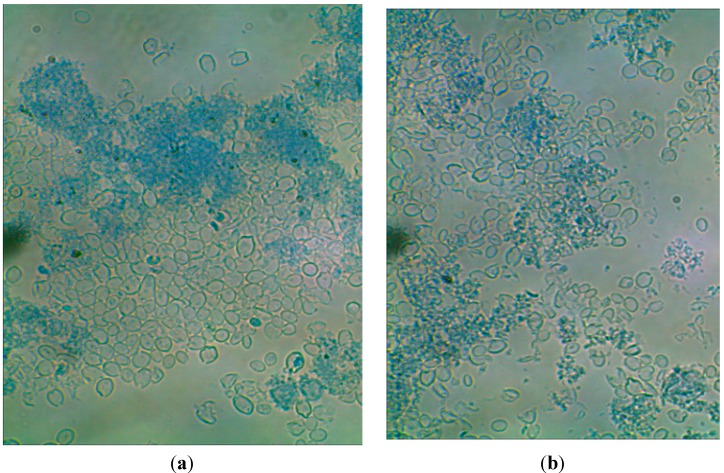
*S. cerevisiae* cell wall preparations after cells disruption with the use of a bead mill; bead Ø—1.0 mm (magn. 600×); (**a**) cells disrupted in deionized water; (**b**) cells disrupted in Tris-HCl buffer.

**Figure 6:**
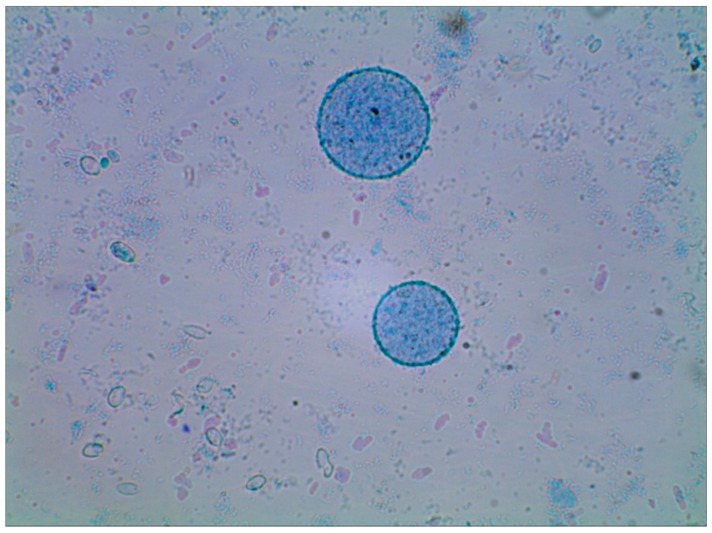
Aggregates of preparations of *S. cerevisiae* yeast cell walls after cells disruption with the use of a bead mill and beads with Ø = 1.0 mm (magn. 600×).

The microscopic observation of the preparations presented in Figure 5 confirmed that homogenization in the bead mill results in disruption of yeast cell wall continuity, which in turn contributed to the effective release of intracellular components. Cell disintegration occurred simultaneously—as evidenced by visible smaller fragments of cell walls of damaged cells. Likewise in studies by Hunter* et al*. [21] and Zechner-Krpan* et al*. [29], formation of aggregates of yeast cell walls polymers could be observed, which indicated the presence of hydrophobic particulated β-glucans. These structures attained a spherical shape with diameters of *ca.* 12 to 70 µm (Figure 6). However, in the mentioned studies [21,29] the aggregates occurred after alkali extraction of β*-*glucan from cell walls.

The effectiveness of disrupting forces, acting during disruption of dead cells (homogenization of cells firstly exposed to autoclaving) was significantly lower compared to the process conducted with live biological material. This allows hypothesizing that the shear forces acting during homogenization in a bead mill more easily damaged these cells whose walls were characterized by some stress and rigidity. Probably after autoclaving the yeast cell wall was already losing its rigidity and as a result the successive stage of mechanical disruption did not contribute to effective removal of cytosol components. A similar dependency was observed for the cells subjected to sonication. However, Middelberg [13] explained that mild heat treatment primarily kill the cells what may lead to smaller, toughened cells, thus making subsequent mechanical disruption less efficient. In turn, Magnani* et al.* [24], after Liu* et al.* [17], report that the changed in the structure of cell wall after yeast cell autoclaving, resulting in the loss of the mannoproteins layer, potentiated the mechanical process of disruption via sonication.

The cell wall preparations produced as a result of *S. cerevisiae* yeast cell exposure to ultrasound in water contained *ca.* 48% of total saccharides, *ca.* 44% of proteins and *ca.* 6.4% of β-glucans. The content of β-glucans turned out to be lower by *ca.* 17% compared to that determined in the intact biomass. Sonication probably led to depolymerization of chains of β-glucans and their loss during the process. Magnani* et al.* [24] advanced a similar hypothesis.

In the case of sonication conducted in the buffer, the content of total saccharides, proteins and β-glucans in the preparations reached *ca.* 59%, *ca.* 40% and *ca.* 13%, respectively. Comparing these results with the preparations after sonication in water, it was found that disruption in the buffer only negligibly decreased the protein content in the preparations (*ca.* 4%), whereas the content of β-glucans was twofold higher. It may be speculated that alkaline pH conditions facilitated the loosening of polymer structure under the influence of ultrasound, which was reflected in easier and, thereby, more effective enzymatic hydrolysis of the polysaccharide during determination of β-glucans content. The formation of a partly open structure of a double helix of yeast β(1,3)-glucan after extraction in alkaline conditions was reported by Young* et al.* [32]. Also, the higher content of β(1,3)/(1,6)-glucans in the preparations after yeast sonication at similar contents of proteins and total sugars as in the preparations after bead mill homogenization could result from the loosening of the three-dimensional structure of this polymer under the influence of ultrasound waves. This could facilitate hydrolytic enzyme access to glycosidic bonds in chains of the polysaccharide during determination of β-glucans content with the enzymatic test. The procedure was based on β*-*glucan enzymatic hydrolysis resulted from the activity of *exo*- β (1,3)-glucanase, *endo*-β(1,3)-glucanase and β*-*glucanase.

### 2.3. Spectral Analysis of Cell Wall Preparations Using Fourier Transform Infrared Spectroscopy (FTIR)

**Figure 7:**
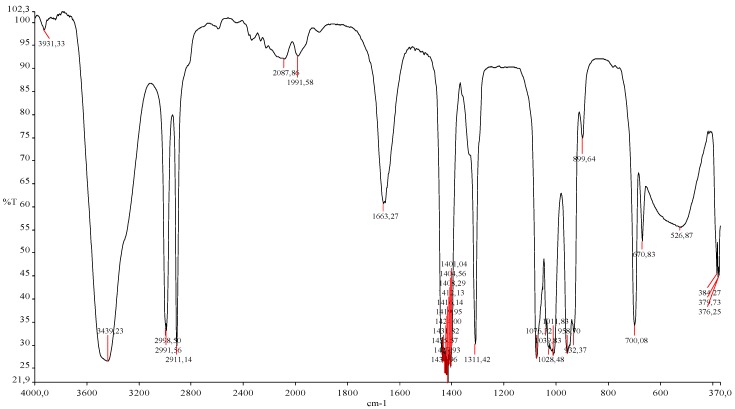
Example of IR spectrum of baker’s yeast cells walls preparation after cell disruption with a bead mill; bead Ø—0.5mm.

The purity of the cell wall preparations can be followed by IR spectral analysis. The IR spectra of the obtained cell wall preparations (Figure 7) show a similar pattern to a baker’s yeast β(1,3)/(1,6)-glucan standard and were in agreement with published data on yeast cell wall polymers [7,33,34]. Due to the similarity of spectra for all studied preparations, only the spectra of cell walls obtained with the bead mill are shown in Figure 7.

The analysed spectra of the prepared cell walls show differences in the intensity of the absorption in the region between 1600–1800 cm^−1^ (with s maximum at approx. 1660 cm^−1^). This was related to the presence of functional groups corresponding to proteins, such as amide bonds and aromatic rings. The presence and differences in protein content in analysed samples were confirmed by the Kjeldahl method and was discussed in a previous section of the paper. However the absorption at the spectral region between 1622–1684 cm^−1^ could also be influenced by the vibrations of nucleic acid base residues [35,36].

The bands between 520 and 1100 cm^−1^ are characteristic of polysaccharides (α*-* and β-glucans, α*-*mannan) [33,37,38]. Simultaneously, the absorption bands located at the spectral region at approx. 700–1090 cm^−1^, observed in all studied cell wall preparations, could be a consequence of genetic material residues and the presence of glycogen in analyzed samples [35,39,40]. For example, guanine and cytosine absorption bands occur between 700 and 784 cm^−1^ while the bands at 800–893 cm^−1^ and 925–1020 cm^−1^ could result from deoxyribose and phosphodiester backbones. The absorption at 1050–1090 cm^−1^ was probably influenced by PO_2_^−^ groups from nucleic acid residues while the one at approx. 1080 cm^−1^ probably was due to glycogen [35,36,39,40]. The absorption bands in the spectral region between app. 2985–3015 cm^−1^ confirmed the presence of lipid residues in the studied cell wall preparations [39].

The shifting of the band maximum position, broadening of the band as well as differences in the intensity of transmittance was noticed in the hydroxyl groups region (3650–3200 cm^−1^) for cell wall preparations obtained by different methods. For example, the maximum band position in the hydroxyl groups region for cell wall preparations obtained with a bead mill (0.5 mm) was approx. 3480 cm^−1^, while for preparations produced by autoclaving in buffer coupled with sonication it was approx. 3437 cm^−1^. The lowest intensity of the hydroxyl groups band was characteristic of preparations obtained via yeast cell sonication. Changes in the hydroxyl stretching region can result from the differences in the polymerization of cell wall polysaccharides (particle size) or intra- and intermolecular hydrogen bond formation/deformation, which influence the helical structure of yeast cell wall polysaccharides [33].

### 2.4. Discussion

The goal of this study was to determine the effectiveness of selected methods of yeast cell disruption on the production of yeast cell wall preparations free, to the greatest possible extent, of the remaining cell components. Simultaneously, the applied methods should not damage the native, biologically-active structure of β-glucan polymers. Four methods of yeast cell disruption were verified in this study: autoclaving, autolysis, disruption with the use of a bead mill, disruption with ultrasound, and their combinations. To this end, *S. cerevisiae* cells were disrupted in 19 experimental systems that are summarized in Table 2 (Experimental Section**)**.

According to the study assumptions, yeast cell wall preparations should be characterized by the the highest possible content of saccharides, and thus β-glucans, at the lowest possible content of impurities, like proteins, the major constituents of *S. cerevisiae* yeast biomass [16]. Hence, the purity of the produced cell wall preparations was determined by assaying the content of total saccharides, β-glucans and proteins. However, the proteins content in cell wall preparations estimated on the basis of total nitrogen quantification, could be overestimated due to the presence of nitrogen from genetic material. According to Hernawan and Fleet [16], RNA, the main nucleic acid in yeast cells, was subjected to rapid and extensive degradation (80%–90%) during autolysis, but some RNA degradation products were probably associated with the cell residues. Hernawan and Fleet [16] and Zhao and Fleet [14] showed that DNA represents only a small proportion of the cell dry weight (less than 1.5%) but DNA occurs in complexes with proteins which may protect this molecule from enzymatic degradation [16].

The total saccharides content was used to compare the purity of cell wall preparations obtained by the different disruption methods. The total sugar in obtained yeast cell wall preparations could be overestimated due to sugars present in genetic material, glycogen or free sugars. However, the soluble saccharides could be extracted in some extent during the cell wall preparation procedures. Further research is needed to determine the content of the mentioned impurities in cell wall preparations.

Contents of individual components in yeast cell wall preparations differ depending on the cell disruption method, conditions of this process (e.g., temperature, pH, duration of disruption, method of preparing yeast suspension, including type of medium and cell concentration). Also the chemical composition of biomass and cell wall of the analyzed yeast strain, and thus culture conditions of these microorganisms, are important. This was confirmed in the works of other authors [7,9,17,19,24,27,28,41]. For instance, the biomass of *S. cerevisiae* yeast used by Suphantharika* et al.* [28] to obtain cell walls via autolysis contained *ca.* 41% of proteins, 55% of total saccharides and 17% of β-glucans, whereas brewer’s yeast investigated by Liu* et al.* [17] contained *ca.* 60% of proteins and 35% of sugars. Freimund* et al.* [20] report that the cell wall preparations of two baker’s yeast strains analyzed in their study and produced via autolysis differed significantly in contents of total sugars and proteins. The autolytic properties of the strain could be decisive here. Cell wall preparations of yeast of the genus *Saccharomyces* were produced via autolysis by other authors [7,17,24,26,28,41,42]. In these cases, the cell walls contained 18%–41% of proteins, 44%–69% of total saccharides and 16%–34% of β-glucans. The coefficient of autolysis determined in studies by Wang* et al.* [41], Liu* et al.* [17] and Magnani* et al.* [18] was lower than stated in our results and fitted within the range of 15%–53%. Differences in these values resulted from, e.g., process conditions.

Homogenization with glass beads was applied by Dallies* et al.* [19], Nguyen* et al.* [27] as well as by Marinescu and Stoicescu [26], but the cell wall preparations varied in the content of proteins (5%–37% d.m.), sugars (*ca.* 56%–93%), and β-glucans (*ca.* 19%–71%). The purity of cell wall preparations obtained by Marinescu and Stoicescu [20] was the most similar to that presented in our work.

Liu* et al.* [12] optimized the process of *S. cerevisiae* yeast disruption with the use of sonication. The authors achieved more effective purification of the cells from proteins after 20 min of sound generation but the power used was 600 W.

Yoshida* et al.* [9] produced *Kluyveromyces marxianus* cell wall preparations as a result of cell autoclaving. These preparations contained *ca.* 41.5% of proteins, compared to *ca.* 46% proteins content in biomass. Irrespective of the yeast genera it confirms our observations that autoclaving is not sufficient to obtain yeast cell wall specimens purified from intracellular components.

When comparing the presented literature data with the characteristics of cell walls produced in our study it was concluded that none of the studied yeast disruption methods allowed us to achieve a protein content lower than 35%. On the other hand, the preparations were characterized by a saccharide content comparable to results presented in literature. The lower contents of β-glucans might, in turn, result from the strain specificity of the applied biological material and the method of β-glucan analysis. It was also possible, that proteins and lipids present in the obtained cell wall preparations made difficult the hydrolytic action of enzymes applied in the enzymatic test for β-glucans analysis. The mentioned compounds cause steric hindrance and as a result not all glycosidic bonds were hydrolyzed and consequently the β-glucans content would be underestimated.

## 3. Experimental Section

### 3.1. Biological Material

The experimental material was baker’s yeast (*Saccharomyces cerevisiae*) with a dry matter content of *ca.* 28%. Such a biological material allowed us to achieved comparable concentrations of yeast dry matter in all suspensions.

### 3.2. Preparation of Yeast Suspension for Disruption

A yeast cell suspension (100 g) with a concentration of *ca.* 5% d.m. was prepared in sterile deionized water (pH 5.0) or in sterile 10 mM Tris-HCl buffer (pH 8.0). Water was deionized in a Direct-Q 3UV-R apparatus (Millipore, Molsheim, France) and sterilized (121 °C/20 min, HICLAVE HG-80 autoclave, HMC Europe, Tuessling, Germany). In the case of autolysis, analyses were additionally carried out in water with pH 7.0. Acidity was adjusted as needed using NaOH or HCl solutions. Before the disruption processes the prepared yeast cells suspensions were stirred for 10 min on a Barnstead/Lab-Line E-class shaker at a frequency of 100 rpm.

### 3.3. Procedures for Yeast Cells Disruption with the Selected Methods

One experimental series included disruption of *S. cerevisiae* cells in 10 or nine variants of the experimental systems, depending on whether disruption was conducted in deionized water or in Tris-HCl buffer. The experimental systems and their parameters are summarized in Table 2.

#### 3.3.1. Autoclaving Process

Suspensions of yeast cells (95 g) in flat-bottomed flasks (volume 500 mL) were autoclaved at a temperature of 115 °C for 10 min in the HiClave HG-80 autoclave [9]. The total time of samples retention in the autoclave was *ca.* 80 min. They were taken out from the autoclave when the internal temperature of the chamber dropped to 80 °C.

#### 3.3.2. Autolysis

Yeast suspensions (95 g) in flat-bottomed flasks (volume 500 mL) were shaken in a Barnstead/Lab-Line E-class shaker at a temperature of 50 °C, at the speed of 200 rpm for 24 h. The autolysis-inducing factor was temperature [7,27].

#### 3.3.3. Homogenization in a Bead Mill

The procedure was based on Kath and Kulicke [23] with modifications. Yeast cells were disintegrated with the use of a bead mill (Bead-Beater type GB26 homogenizer, BioSpec Products, Bartlesville, OK, USA) with the power of 400 W. The total working volume of the mill tank was *ca.* 300 mL. Zirconium-glass beads (BioSpec Products) with diameters of 0.5 or 1.0 mm were used in the experiment. The beads were added to the volume of 170 cm^3^, which constituted 57% of the working volume of the tank.

**Table 2:** Parameters of experimental systems used in the study (* Abbr. means abbreviation of a given method).

Type of Method	Experimental System	Preparation of Suspensions			
		Cell Suspensions in Deionized Water	Abbr. *	Cell Suspensions in 10 mM Tris-HCl Buffer (pH 8.0)	Abbr. *
Individual methods	Autoclaving	115 °C, 10 min	**a**	115 °C, 10 min	**aB**
	Autolysis	pH 5.0, 50 °C, 24 h, 200 rpm	**al_pH5**	50 °C, 24 h, 200 rpm	**alB**
	Autolysis	pH 7.0, 50 °C, 24 h, 200 rpm	**al_pH7**		
	Sonication	4 × 5 min/2 min, pulser 80%, power 80%	**s**	4 × 5 min/2 min, pulser 80%, power 80%	**sB**
	Bead mill	Ø 0.5 mm, 5 × 3 min/3 min	**m 0.5**	Ø 0.5 mm, 5 × 3 min/3 min	**m 0.5B**
	Bead mill	Ø 1 mm, 5 × 3 min/3 min	**m 1.0**	Ø 1 mm, 5 × 3 min/3 min	**m 1.0B**
Coupled methods	Autoclaving	115 °C, 10 min	**a_s**	115 °C, 10 min	**a_sB**
	Sonication	4 × 5 min/2 min, pulser 80%, power 80%		4 × 5 min/2 min, pulser 80%, power 80%	
	Autoclaving	115 °C, 10 min	**a_m**	115 °C, 10 min	**a_mB**
	Bead mill	Ø 0.5 mm, 5 × 3 min/3 min		Ø 0.5 mm, 5 × 3 min/3 min	
	Autolysis	pH 5.0, 50 °C, 24 h, 200 rpm	**al_m**	pH 5.0, 50 °C, 24 h, 200 rpm	**al_mB**
	Bead mill	Ø 0.5 mm, 5 × 3 min/3 min		Ø 0.5 mm, 5 × 3 min/3 min	
	Autolysis	pH 5.0, 50 °C, 24 h, 200 rpm	**al_s**	pH 5.0, 50 °C, 24 h, 200 rpm	**al_sB**
	Sonication	4 × 5 min/2 min, pulser 80%, power 80%		4 × 5 min/2 min, pulser 80%, power 80%	

Consideration was given to the manufacturer’s recommendation, according to which the load of beads should reach minimally 1/2 and maximally 3/4 of tank volume. After adding 95 g of the yeast suspension to the tank, the experimental system was assembled. Throughout the process, the tank was cooled with an ice bath jacket. Disruption included five cycles each consisting of 3 min of homogenizer work and a 3 min break. During cell wall preparation recovery after disruption, the beads were washed 3-times with 50 cm^3^ of deionized water and the filtrate was centrifuged.

#### 3.3.4. Sonication

The process was based on Liu* et al.* [12] with modification. Sonication was carried out using an Omni Ruptor 4000 homogenizer (OMNI International, Kennesaw, GA, USA) and a titanium terminal (19.1 mm in diameter) generating ultrasounds. The yeast suspension was placed in a water bath with ice, maintaining a stable level of terminal submersion (*ca.* 2.5 cm). Disruption included four cycles consisting of 5 min of sonication and a 2 min break. During one cycle of sonication 80% of process time was the period of impulse generation. The homogenizer was working with 80% of its maximum working power (300 W). The ultrasound frequency was 20 kHz.

### 3.4. Microscope Photos

The effectiveness of yeast cell disruption and their purification from cytosol components was evaluated microscopically immediately after cell disruption. Preparations were stained with methylene blue. Microscopic observations were carried out under an MB300 light microscope MB300 (OPTA-TECH, Warsaw, Poland) at 600× magnification. Photos of the preparations were taken using OptaView 7 camera and software (OPTA-TECH).

### 3.5. Cell Wall Preparations

After disruption, precipitates of damaged cells were collected by centrifugation of the suspensions (5000 *g*/4 °C/5 min). The resultant cell wall preparations were prepurified from cytosol components before further analyses. To this end, 30 mL of distilled water were added to the pellet obtained from the last centrifugation. Next, contents of test tubes were vortexed and centrifuged (5000 *g*/5 min/4 °C), and the supernatant was decanted. Next, the precipitate was washed three times with 30 mL of NaCl solution with increasing concentrations, *i.e*., 17 mM, 34 mM and 85 mM. The last precipitate washes were performed with deionized water (two times with 30 mL each). Each time, the samples were centrifuged under the centrifugation parameters stated above. The resultant preparations were dried at 80 °C for 24 h, and ground in an MKM 6003 grinder (Bosch, Munich, Germany).

### 3.6. Determination of the Percentage of Solubilised Material

The percentage of solubilised material for each studied method was determined on the basis of dried matter content before and after each disruption process. The following formula was used for calculation [17]:

(1) $$R\left(\%\right)=\frac{M_{0}-M}{M_{0}}\;*\;100\%$$

where: R—percentage of solubilised material, M_0_—dry matter in 1 mL of the suspension before disruption, and M—dry matter in 1 mL of the suspension left after the yeast cell disruption.

Dried matter content was determined in yeast cell suspensions before and after disruption. Determination of dry matter content of a crude preparation of yeast cell walls required rinsing the precipitates obtained after centrifugation (5000 *g*/4 °C/5 min, Eppendorf 5810R Centrifuge) of the cell residues damaged in the disruption process a few times with water and NaCl solutions. To this end, disruption residues were washed with 30 mL of distilled water under intensive stirring on a vortexer, and then the samples were centrifuged using the parameters given above. Afterwards, 30 mL of NaCl solution was applied at an increasing concentration, *i.e*., 17 mM, 34 mM and 85 mM; each time the samples were stirred and centrifuged. The last washes of the precipitates were conducted with deionized water (two times with 30 mL) to desalt the samples. Next, the samples were dried (105 °C to a constant weight, *i.e*., for *ca.* 24 h).

### 3.7. Determination of the UV Absorption of Yeast Cell Solubilised Material

The method was based on Liu *et al.* [12] with modifications. Solubilization of the studied yeast intracellular material was determined on the basis of the increase of sample absorbance read at 260 and 280 nm after yeast disruption. For that purpose 1 mL of yeast cell suspension was taken before and after cell disruption. Samples were centrifugated (5000 *g*/5 min, MiniSpin plus, Eppendorf, Hamburg, Germany). Obtained supernatants were diluted eight times using water or Tris-HCl buffer before measurements. Absorbance was read at 260 and 280 nm (UV-1800 UV/VIS, Rayleigh, Beijing, China) against water or buffer. The increase of absorbance of samples at each wavelength was calculated as a difference between the absorbance of samples after and before a particular disruption process.

### 3.8. Characteristics of Produced Cell Walls Preparations

#### 3.8.1. Total Saccharides Content

The content of total saccharides was determined with a colorimetric method with DNS at the wavelength of 540 nm (UV-1800 UV/VIS, Rayleigh) [7]. Reducing sugars were expressed as glucose. Before determination, cell wall polymers (app. 20 mg) were subjected to acidic hydrolysis (72% H_2_SO_4_, 95 °C/4 h) in a water bath (Memmert WNB14, Schwabach, Germany) [19]. The temperature of hydrolysis was modified from 100 °C to 95 °C without influencing the process efficiency. The content of saccharides was calculated using a standard curve prepared for glucose (y = 2.8217 × −0.0714, R^2^ = 0.9994).

#### 3.8.2. β(1,3)(1,6)-Glucans Content

The content of β(1,3)/(1,6)-was determined using an Enzymatic Yeast β-Glucan Kit (K-EBHLG 03/13 Megazyme, Bray, Ireland) following the procedure recommended by the manufacturer [33,43]. Briefly, yeast β-glucan present in cell wall preparations was solubilised/hydrated in 2 N KOH during incubation in an ice water over a magnetic stirrer. After 30 min the solution was subsequently adjusted to pH 4.0–4.5 with 1.2 M sodium acetate buffer. Then the slurry was incubated for 16 h at 40 °C with *Glucazyme* enzyme mixture (*exo*-1,3-β-glucanase, *endo*-1,3-β-glucanase, β-glucosidase and chitinase suspension). After dilution and centrifugation an aliquot was removed for determination of glucose with the GOPOD reagent consisting of glucose oxidase, peroxidase and 4-amino-antipyrine in buffer prepared using *p*-hydroxybenzoic acid and sodium azide (0.4% w/v, pH 7.4). The free glucose was oxidized by glucose oxidase to gluconic acid and to hydrogen peroxide. Next, the hydrogen peroxide was reduced by peroxidase while the 4-aminoantipyrine was oxidized to coloured product of which the absorbance was measured. The absorbance of samples and a standard of D-glucose (1.5 mg/cm^3^) were measured at 510 nm against a reagent blank. Calculations of β-glucan content were based on the formula:

(2) $$\beta \left(1,3\right)\left(1,6\right)-glucan\;\left(\%\;w/w\right)=\Delta E\times F\times \frac{12.04}{0.1}\times \frac{100}{W}\times \frac{1}{1000}\times \frac{162}{180}$$

where: ΔE—absorbance read against blank, F—conversion from absorbance to µg (150 µg of d-glucose) standard divided by GOPOD absorbance of this 150 µg, 12.04/0.1—volume correction (0.1 mL taken from 12.04 mL), 1/1000—conversion from µg to mg, W—weight of sample analysed in mg, 100/W—factor to present β(1,3)(1,6)-glucan as a percentage of sample weight, 162/180 factor to convert from free d-glucose to anhydro-d-glucose as occurs in β(1,3)(1,6)-glucan.

#### 3.8.3. Total Nitrogen Content

Total nitrogen content in the analyzed samples was determined with the Kjeldahl method (Büchi mineralization and distillation units, Büchi Labourtechnik, Flawil, Switzeland). Nitrogen content was expressed per crude proteins using a conversion factor of 6.25 [17].

#### 3.8.4. FTIR Spectral Analysis

FTIR spectra of yeast cell wall preparations were determined with a System 2000 instrument (Perkin Elmer, Waltham, MA, USA) in DMSO solutions at a resolution of 2 cm^‒1^. KBr cuvettes with an absorptive surface thickness of 0.22 mm were used. The spectral region was 4000–350 cm^‒1^. Spectra were analyzed with the Spectrum v. 2.00 software. A baker’s yeast β-glucan standard (>98%, G5011 Sigma Chemical CO., Saint Louis, MO, USA) was used for comparison. The concentration of the cell walls preparations in DMSO was app. 7 mg/mL.

### 3.9. Statistical Analysis of Results

The significance of differences between results obtained in the study was analyzed using Tukey’s test and Statistica v. 10.1 software (StatSoft Polska Sp. z o.o., Krakow, Poland), at a significance level of α = 0.05.

## 4. Conclusions

In summary, the use of different methods for yeast cell disruption had a significant effect upon β-glucan content in the resulting cell wall preparations. It was linked with the various degrees of purification of the preparations. Among the analyzed methods, the most promising results were achieved by using a bead mill as a single method, autolysis coupled with this type of homogenization as well as autolysis with ultrasound. Cell wall preparations produced with these methods were characterized by the lowest proteins content while the content of total saccharides and β-glucans were the highest. However, considering the time needed for the production of cell wall preparations the fast method of yeast cell homogenization in a bead mill was identified as the most interesting option. This method is also more valuable for general isolation precedures because it allows one to eliminate the different autolytic activity of various yeast strains. Results obtained for the preparations produced with the use of ultrasound demonstrate depolymerization of β-glucans and, thereby, losses are likely to occur during sonication.

The poorest release of intracellular components was observed as a result of cell autoclaving. The mechanical methods had no significant effect on the efficiency of this kind of disruption. Besides, the effectiveness of disrupting forces acting during disruption of dead cells (cells firstly exposed to autoclaving) was significantly lower compared to the process conducted with live biological material. It was probably due to the fact the shearing and disrupting forces that act during homogenization in a bead mill or during sonication damage more easily those cells whose wall is characterized by some turgor, what means some stress and rigidity. After autoclaving, the yeast cell walls were probably losing their rigidity and as a result of this the subsequent mechanical disruption stage did not contribute to effective removal of cytosol components. It was also possible that heat treatment primarily killed the cells which would lead to smaller, toughened cells, thus making any subsequent mechanical disruption less efficient.

It was additionally observed that treating the yeast biomass with hot water led to the loss of part of the β-glucans soluble under such conditions. In order to obtain purified isolates of β-glucans from yeast cell wall preparations produced with the aforementioned methods it is necessary to conduct successive stages of purification including deproteinazation, defatting, as well as removal of genetic material and glycogen residues.
